# Circulating biomarkers in the diagnosis and management of neuroendocrine neoplasms

**DOI:** 10.1530/EO-25-0115

**Published:** 2026-10-28

**Authors:** Angie S Xiang, Emma Boehm, Sunita M C De Sousa, Roderick J Clifton-Bligh, Richard W Tothill

**Affiliations:** ^1^ St Vincent’s Hospital, Melbourne, Australia; ^2^ Peter MacCallum Cancer Centre, Melbourne, Australia; ^3^ Department of Clinical Pathology, University of Melbourne, Melbourne, Australia; ^4^ Collaborative Centre for Genomic Cancer Medicine, University of Melbourne, Melbourne, Australia; ^5^ Royal Adelaide Hospital, Adelaide, Australia; ^6^ Royal North Shore Hospital, Sydney, Australia; ^7^ Sir Peter MacCallum Department of Oncology, University of Melbourne, Melbourne, Australia

**Keywords:** biomarker, neuroendocrine tumours, proteomics, neoplasia, gene expression

## Abstract

Neuroendocrine neoplasms (NENs) comprise a broad family of tumours arising from the diffuse neuroendocrine system and endocrine glands and vary widely in clinical behaviour ranging from indolent to highly aggressive. Established monoanalyte biomarkers, including chromogranin A and NEN-derived hormones, have clinical utility but they also have some major limitations including non-specificity for tumour origin, variable sensitivity between NEN subtypes, inconsistent correlation with prognostic factors including metastasis, and can be falsely elevated in common non-NEN pathologies. The expansion of investigative NEN biomarkers through advancing omics technologies and computational methods holds potential to improve diagnosis and prognostication and to aid in therapy selection or trial allocation. This review will outline established and evolving NEN circulating biomarkers and their roles in clinical practice.

## Introduction

Neuroendocrine neoplasms (NENs) are a diverse group of malignancies that all share neuroendocrine differentiation and arise from the diffuse neuroendocrine system (e.g. gastrointestinal and bronchopulmonary NENs) or the classic endocrine glands (e.g. pancreatic, adrenal, thyroid, parathyroid, and pituitary NENs) (1). In addition to the tissue of primary origin, NENs are pathologically classified by differentiation and grade, and are broadly divided into either well-differentiated neuroendocrine tumours (NETs; grades 1–3) or poorly differentiated neuroendocrine carcinomas (NECs; all grade 3) (2). NET behaviour is highly heterogeneous: although these cancers are often indolent, they can be aggressive and metastatic, so accurate prognostication to inform management and surveillance is a clinical imperative (3, 4, 5).

The current approach for the diagnosis and surveillance of NENs involves high-resolution imaging with computed tomography (CT), magnetic resonance imaging (MRI), and positron emission tomography/computed tomography (PET/CT); biochemical assessment of tumour secretory products; and histopathological analysis (3, 4, 5). Functional PET/CT imaging is generally performed with somatostatin analogue tracers (e.g. Gallium-68 DOTATATE) which are highly sensitive and specific for NETs, serving an important role in tumour staging and interval disease monitoring, although it offers limited additional prognostic information. Meanwhile, fluorodeoxyglucose F-18 (18F-FDG) avidity can delineate tumour aggressiveness and predict post-treatment outcomes in high-grade NENs; however, it is rarely positive in lower-grade NETs (6, 7). The use of cross-sectional cancer imaging is associated with several limitations: access can be limited in many geographical regions; repeated testing poses a significant financial, time, and psychological burden on patients; and there are risks with lifetime cumulative radiation exposure (8). Furthermore, established blood-based tests (e.g. chromogranin A and hormones) have some clinical utility but can be limited in sensitivity and specificity depending on NEN subtype and the functionality of a patient’s tumour, while also having limited value to direct therapy. The lack of readily accessible, affordable, and accurate non-invasive tests for NENs is of particular importance for patients with heritable NET syndromes such as multiple endocrine neoplasia type I (MEN1), who require frequent multimodal screening and surveillance to evaluate for several tumour types (9).

Identification of additional NEN blood biomarkers with clinical utility has been a longstanding unmet need and a priority for research (10). Emerging tumour-derived biomarkers including messenger RNAs (mRNAs), microRNAs (miRNAs), long non-coding RNAs (lncRNAs), circulating tumour DNA (ctDNA), circulating tumour cells (CTCs), extracellular vesicles (EVs), and tumour-related proteins/peptides have been investigated as biomarkers to inform NEN patient care, from early stage to metastatic disease settings (Fig. 1, Table 1). This review will examine these current and evolving blood-based biomarkers and technologies showing promise for NEN patients.

**Figure 1:**
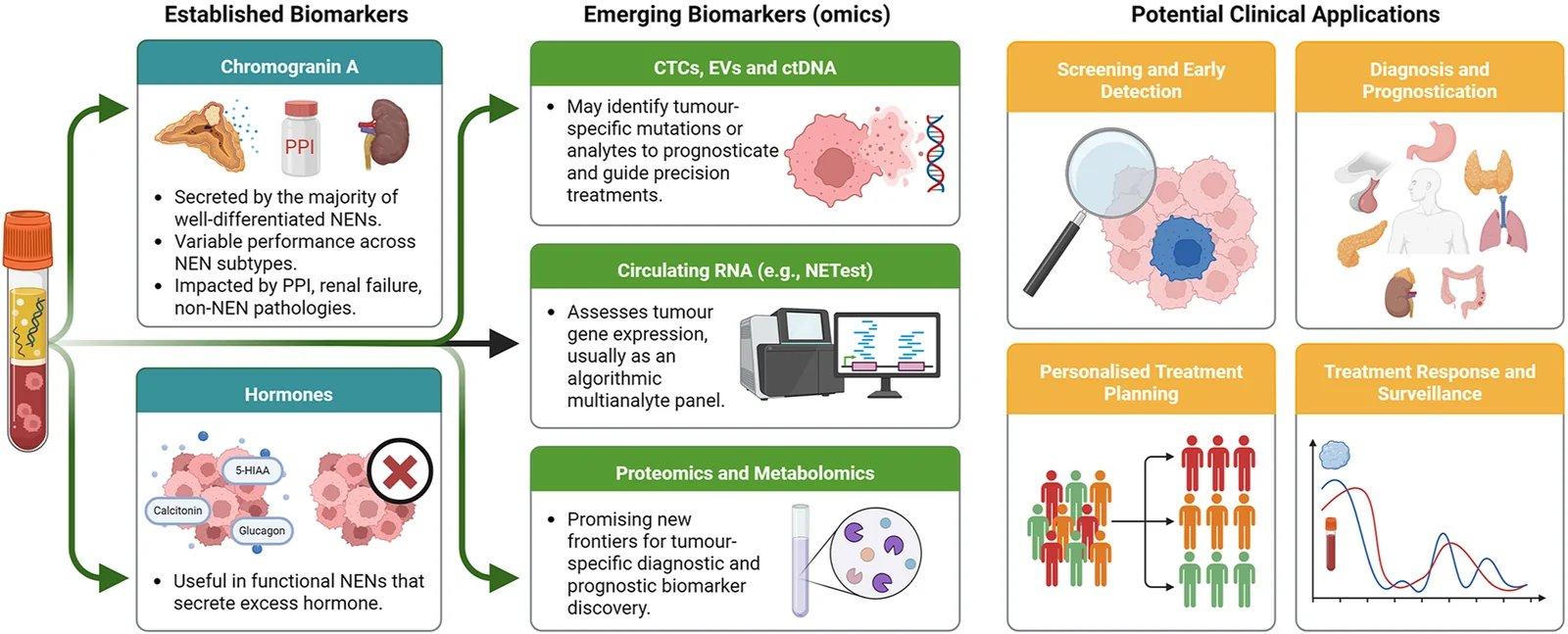
Established and emerging circulating blood-based biomarkers for neuroendocrine neoplasms (NENs) and potential clinical applications. Several NEN-related analytes can be detected in circulation as potential biomarkers with established or potential clinical utility. Established biomarkers are predominantly protein and small molecule monoanalytes (e.g. chromogranin A, NEN hormones). Other emerging NEN-related biomarkers found in blood circulation include nucleic acids (RNA and DNA), circulating tumour cells (CTCs), extracellular vesicles (EVs), proteins or peptides, and metabolites. Potential clinical applications for these novel NEN circulating biomarkers include disease screening and early detection, tumour diagnosis and subtyping, personalised treatment selection, monitoring for treatment response or resistance, and surveillance. PPI, proton pump inhibitor; 5-HIAA, 5-hydroxyindoleacetic acid; ctDNA, circulating tumour deoxyribonucleic acid; RNA, ribonucleic acid. Created in BioRender.com.

**Table 1:** Summary of established and investigational circulating blood-based biomarkers for neuroendocrine neoplasms (NENs).

Analyte class	Biomarker	Indication/potential indication	Limitations
Non-hormone protein monoanalytes	**Granins (e.g. chromogranin A) (3, 11, 12)**	Disease monitoring in some NENs (generally gastroenteropancreatic NETs)	Variable sensitivity depending on NEN subtype Low specificity with interference by comorbid conditions (e.g. renal dysfunction, heart failure) or medication use (e.g. proton pump inhibitors) Does not inform NEN subtype/primary tumour location No prognostic value
	**Neuron-specific enolase**	Disease monitoring in some NENs (generally atypical carcinoids and bronchopulmonary NECs) (3) Under investigation as a prognostic marker in clinical trials (13, 14)	Low sensitivity and specificity Does not inform NEN subtype/primary tumour location Susceptible to pre-analytical error from improper sample handing
Hormones and bioactive peptides (3, 5, 15, 16, 17, 18, 19)	**Serotonin, 5-HIAA**	Carcinoid syndrome	Negative in non-functioning tumours Variable specificity to NEN subtype/primary tumour location depending on biomarker Some biomarkers are prone to pre-analytical interference (e.g. test timing, fasting status, medication use, comorbid non-NEN disease) Limited prognostic value
	**Gastrin**	Zollinger–Ellison syndrome/gastrinoma	
	**Insulin, pro-insulin, C-peptide**	Insulinoma	
	**Glucagon**	Glucagonoma	
	**VIP**	VIP-oma	
	**Somatostatin**	Somatostatinoma	
	**Pancreatic polypeptide, β-hCG, a-fetoprotein**	Pancreatic NETs (both functional and non-functional)	
	**Catecholamines, metanephrines**	Phaeochromocytoma and paraganglioma	
	**Calcitonin**	Medullary thyroid cancer and pancreatic NET	
	**Prolactin, GH, IGF-1, ACTH, cortisol, FSH, LH, oestradiol, testosterone, TSH, T3, T4, α-subunit**	Pituitary NETs	
	**PTHrP, ACTH, CRH, GHRH, copeptin**	Paraneoplastic syndromes mainly from ectopic lung or pancreatic NET Ectopic ACTH and SIADH can be associated with NEC	
RNA	mRNA – NETest (20, 21, 22)	Diagnosis, prognostication and disease monitoring in mixed NETs (greatest evidence in lung and gastroenteropancreatic NET) (21, 22) Predict response to peptide receptor radionuclide therapy in lung and gastroenteropancreatic NET (23)	Variable sensitivity and specificity reported between studies Does not inform NEN subtype/primary tumour location Poor accessibility (laboratory-developed test)
	miRNA and lncRNA	Emerging research domain – several miRNAs and lncRNAs are reported to be differentially expressed in NEN patients versus controls, with some demonstrating potential prognostic value	No markers have demonstrated NEN specificity Requires substantial further exploration and validation
DNA	ctDNA and cfDNA	Diagnosis, prognostication, and disease monitoring in some (typically high-grade) NENs (24, 25, 26) Potential to identify therapeutically actionable mutations in the absence of standard tissue biopsy (18)	Low utility in low-grade, early-stage NETs (26, 27)
Tumour cells and extracellular vesicles	Circulating tumour cells	Prognostic marker if present (28, 29) Potential to identify therapeutically actionable mutations in the absence of standard tissue biopsy	Low sensitivity particularly in low-grade, early-stage NETs
	Extracellular vesicles	Emerging research domain – small extracellular vesicles are reported to be increased in pancreatic NET patients versus controls (30)	Requires substantial further exploration and validation
Multianalyte proteomic biomarkers		Emerging research domain – numerous proteins are reported to be differentially expressed in NEN patients versus controls (31, 32)	
Metabolomics		Emerging research domain – metabolomic profiles are reported to differ between NEN patients versus controls (33, 34) Potential to inform pathogenic variant in phaeochromocytomas and paragangliomas	

Summary of currently available established (i.e. endorsed by consensus guidelines; indicated in bold) and investigational biomarkers for NENs.

NET, neuroendocrine tumour; NEC, neuroendocrine carcinoma; 5-HIAA, 5-hydroxyindoleacetic acid; VIP, vasoactive intestinal peptide; hCG, human chorionic gonadotrophin; GH, growth hormone; IGF-1, insulin-like growth factor 1; ACTH, adrenocorticotropic hormone; FSH, follicle-stimulating hormone; LH, luteinising hormone; TSH, thyroid-stimulating hormone; T3, triiodothyronine; T4, thyroxine; PTHrp, parathyroid hormone related peptide; CRH, corticotrophin-releasing hormone; GHRH, growth-hormone-releasing hormone; SIADH, syndrome of inappropriate antidiuretic hormone secretion; mRNA, messenger RNA; miRNA, microRNA; lncRNA, long non-coding RNA; ctDNA, circulating tumour DNA; cfDNA, cell-free DNA.

## Established biomarkers: measuring hormone secretion and monoanalyte testing

### Chromogranin A (CgA)

The granin family of glycoproteins forms major constituents of the dense-core secretory granules characteristic of neuroendocrine cells. Granins play a regulatory role in secretory granulogenesis, protein sorting, granule maturation, and condensation essential to the controlled secretion of hormones and neurotransmitters (35). Granins and their post-translational peptides also impact a range of other biological activities including vasodilation, cell death, and metabolism (35).

CgA is the most well-characterised granin, having been recognised as a biomarker of NEN since the late 1970s, and is the only granin routinely assessed as part of guideline-directed practice (3, 4, 5). It is the most widely expressed granin among neuroendocrine cells according to current research and is commonly expressed among well-differentiated NETs except for some lactotroph pituitary NETs and pancreatic NETs (35). In general, blood serum or plasma CgA is considered the most sensitive granin for the detection of NETs, although test sensitivity is highly dependent on tumour origin (3). One study of patients with metastatic carcinoid and gastroenteropancreatic NETs demonstrated elevated plasma CgA levels in 99% of cases, compared with 88% for chromogranin B (CgB), 6% for chromogranin C (CgC), and 46% for pancreastatin (36). Meanwhile, in another study of 211 patients with various NETs, serum CgA was high in 100% of gastrinoma cases despite low tumour volumes (37). The same study demonstrated elevated plasma CgA concentrations in 89% of phaeochromocytoma, 80% of carcinoid tumour, 69% of non-functioning pancreatic NET, and 50% of medullary thyroid carcinoma cases, in comparison to 13% of pituitary NET, 10% of insulinoma, and 8% of paraganglioma cases, thus highlighting its lack of tissue specificity and limitations in diagnostic utility (37). CgA also has limited utility in poorly differentiated NECs, whereby patients typically have much lower levels (3, 4). In patients who do have an elevated CgA and advanced NET, levels correlate with tumour volume and can be, therefore, used as a biomarker of systemic therapy response and in post-therapy surveillance (37).

It is well recognised that plasma CgA levels can be elevated in a range of non-NEN conditions including renal dysfunction, heart failure, and type A gastritis, as well as with proton pump inhibitor treatment; therefore, CgA must be interpreted with caution in such patients (3). Interestingly, although CgB is not reported to have a major diagnostic advantage over CgA, CgB levels are not elevated in patients treated with proton pump inhibitors (38). Further research is needed to assess the complementarity of different granins or any potential benefit in the detection of multiple granins in the blood of NEN patients.

### Neuron-specific enolase

Neuron-specific enolase (NSE) is an isoenzyme of the glycolytic enzyme enolase and is expressed in neurons and peripheral neuroendocrine cells (38). NSE is not used in routine clinical practice; however, it has been explored as a prognostic biomarker in clinical trials. In the RADIANT-1 trial of 150 participants with well-differentiated (low- to intermediate-grade) advanced pancreatic NET treated with everolimus, those with an elevated baseline NSE had a shorter median progression-free survival (7.8 months) and overall survival (approximately 14 months) than those with non-elevated levels (progression-free survival 12.3 months; overall survival 24.9 months) (13). In another observational study of 592 patients with sporadic metastatic gastroenteropancreatic NETs, median overall survival was 161.8 months in the NSE ≤ 1× upper limit of normal (ULN) group, compared with 27.8 months in the NSE > 3× ULN group (14).

### Hormones and bioactive peptides in functional NEN

Assessment of excess hormones and bioactive peptides secreted by the subset of so-called functional NENs can indicate the primary tissue of origin (e.g. prolactin excess indicating a pituitary prolactinoma), provide prognostic information (e.g. functional pituitary adenomas being typically less aggressive than their biochemically ‘silent’ counterparts (39)), indicate risk of comorbidities (e.g. 5-hydroxyindoleacetic acid (5-HIAA) levels being associated with risk of carcinoid heart disease (3, 38)), serve as a biomarker for disease screening or monitoring, and guide treatment selection. Moreover, hormone excess can produce characteristic clinical syndromes or endocrine emergencies, leading to patients with functional NENs coming to medical attention and potentially being diagnosed at an earlier stage than clinically silent, non-functional disease. Endocrine assessment of functional NENs is nuanced as many hormones are prone to pre-analytical interference through test timing, fasting status, concomitant medication use, and comorbid non-NEN disease states. Moreover, some hormones require dynamic testing (i.e. using pharmacologic or physiologic stimuli to provoke or suppress secretion) to fully elucidate pathological behaviour. Despite generally high sensitivity and specificity, especially when compared with CgA, non-specific clinical presentations and issues with endocrine dynamic testing performance and interpretation can still result in significant diagnostic delays (40). Identification of a functional syndrome is not equivalent to a NEN diagnosis, and adjunctive imaging studies for disease localisation and staging, plus histological characterisation of disease, are still required. Details regarding the endocrine manifestations and assessment of functional NENs have been reviewed in detail by others, and consensus recommendations outlined in subtype-specific international NEN guidelines, and thus will not be further discussed in this review (3, 5, 15, 16, 17, 18, 19).

## Investigational biomarkers: omics and non-invasive evaluation of disease biology

Conventional biochemistry has limited diagnostic utility and neither provides specific information about tumour biology to enable accurate prognostication nor aids in treatment decision-making. Multiple omics-based approaches are, therefore, under investigation.

### RNA

#### Messenger RNA (mRNA): NETest

Assessment of tumour-derived mRNA aims to reveal tumour-specific gene expression profiles, including alterations resulting from epigenetic modifications, microRNA (miRNA) interactions, or other mechanisms. The NETest is a multianalyte algorithmic mRNA biomarker test developed by Modlin and colleagues for diagnostic, prognostic, and predictive applications across broad NET subtypes. It involves a two-step protocol of mRNA isolation from an EDTA peripheral whole blood sample, followed by real-time polymerase chain reaction (RT-PCR) to assess the expression of 51 genes (22). Gene expression is extrapolated to the activity of investigator-defined ‘omes’, and the output is presented as a NET activity index (NETest score) ranging from 0 to 100. This methodology has shown low inter- and intra-assay variability, although analysis is performed in a single laboratory, as well as high reproducibility in consecutive daily samples collected by a large number of clinicians across multiple international centres (22). There is some discrepancy between studies regarding the cut-offs for NETest scores – one common interpretation is that a NETest score of ≤20 is normal, 20–40 represents low risk stable disease, and >40 confers high risk progressive disease (20, 21).

The NETest has demonstrated high sensitivity and specificity (exceeding 90% for both) for the diagnosis of NETs in several trials with a meta-analysis defining a pooled area under the receiver operating characteristic curve (AUROC) of 0.954 (21). In a large prospective multinational study of 1,684 patients with NET, NETest results were concordant with tumour grade, extent, activity, and imaging findings (41). Moreover, NETest scores following surgery were reflective of surgical intent (palliative versus curative surgery) and predictive of post-operative recurrence following curative-intent surgery. The NETest has demonstrated superiority over CgA in NET diagnostic accuracy across several studies and is not known to be affected by the aforementioned non-NEN conditions and medications that can confound CgA levels (20, 21, 41). In a comparative cohort of 1,270 patients with matched serial NETest and CgA measurements, the NETest was also superior for tumour grading, identification of metastatic disease, and evaluation of response to surgery (41).

However, a more recent study that included participants with non-neuroendocrine GIT malignancies as controls reported a lower NETest specificity of 67% (20). This is likely related to the non-NET-specific nature of many genes comprising the NETest panel – including those representing the fibrosome, inflammasome, apoptome, and proliferome – which can be elevated in various other inflammatory and proliferative states (42). Although the performance of the NETest has been demonstrated in cohorts containing patients with common comorbidities including gastroesophageal reflux disease, chronic obstructive pulmonary disease, and hypertension (41), these findings highlight the need for further validation of the NETest in diverse clinical populations. Beyond low specificity for NET in some cohorts, other limitations of the NETest include that it neither identifies actionable therapeutic targets nor discriminates between different NET types which limits its diagnostic utility, particularly in patients with heritable NET syndromes who are at risk of several different NETs and potentially synchronous tumours. Furthermore, the NETest could not predict metastatic progression in MEN1 carriers (43). Finally, the feasibility for widespread implementation in terms of cost, logistics, and availability/accessibility of this assay is unknown – it is currently administered as a laboratory-developed test (LDT) available solely in one location worldwide. As such, the NETest is not currently recommended as part of standard clinical care in NET society guidelines.

An extension to the NETest, termed the peptide receptor radionuclide therapy (PRRT) predictive quotient (PPQ), has recently been developed, which aims to predict response to PRRT among patients with lung and gastroenteropancreatic NETs, producing a binary output of predicted PRRT responders (i.e. predicted to achieve disease stabilisation or a partial response) versus non-responders. The PRRT-PPQ remains an investigational adjunct at this stage having only been examined in several small cohorts globally (23).

#### MicroRNA (miRNA) and long non-coding RNA (lncRNA)

miRNAs are a group of short (typically 19–25 nucleotides in length) non-coding RNA molecules which function to interact with target mRNAs to regulate post-transcriptional gene expression. miRNAs are detectable intracellularly and extracellularly in biological fluids, where they exert autocrine, paracrine, and/or endocrine functions (38). There is an evolving understanding of the role of miRNAs in NET pathogenesis, which thus far includes the modulation of cellular proliferation, differentiation, apoptosis, immune function, and drug response (38). Unlike mRNAs, extracellular miRNAs are highly stable in biological fluids even when subjected to deleterious conditions following specimen collection and thus represent more logistically feasible clinical biomarkers, although to date they remain firmly within the discovery research phase, with no candidates adequately established for clinical translation (44).

Several circulating NEN miRNA biomarkers have been described, but none are known to be entirely specific for NEN compared with other disease states and can also be expressed in healthy neuroendocrine cells. Many miRNAs that have been evaluated for NET prognostication, including miR-21 (45, 46), miR-29 (44), miR-96 (47, 48), miR-202 (49), and miR-375 (45, 50), are involved in tumourigenesis and are dysregulated in a number of non-NEN malignancies (44, 51, 52, 53). Regarding diagnosis, miR-193b is increased in the tumour and serum of patients with pancreatic NET in comparison with healthy controls (54); however, it is also elevated in those with type 2 diabetes mellitus and impaired glucose tolerance (55). Serum miR-222 is elevated among patients with type 1 gastric NET but does not discriminate from other hypergastrinaemic states such as atrophic autoimmune gastritis (56).

Owing to the lack of NEN specificity of individual miRNAs identified to date, the future paradigm for establishing miRNA as NEN biomarkers may involve the development of a multianalyte and machine learning approach to produce a diagnostic or prognostic score (38). Recently, two groups developed miRNA panels applied to tissue samples to differentiate varying grades of pancreatic NET (57, 58); however, the translatability of such results to blood samples remains unknown. Overall, existing miRNA studies have consistently been limited by cross-sectional design, small cohorts, heterogeneous platforms, and lack of external validation; these barriers must be overcome before the true utility of such biomarkers in NET can be established.

lncRNAs are non-coding RNA molecules typically >200 nucleotides in length which regulate gene expression at multiple levels. lncRNAs have so far been applied to attempt to resolve the diagnostic dilemma of parathyroid adenoma versus carcinoma. Serum levels of lncRNA BC200/BCYRN1 have been observed to be higher in patients with parathyroid carcinoma in comparison to those with parathyroid adenoma (59). Furthermore, to illustrate a potential future role for lncRNA in response assessment and surveillance, circulating BC200 counts, as measured by digital PCR, reduced following parathyroid carcinoma resection, noting that data were only available for three patients (59).

### Circulating tumour DNA (ctDNA) and cell-free DNA (cfDNA)

ctDNA describes tumour-derived DNA fragments found in blood released through multiple mechanisms including apoptosis and active release, whereas cfDNA is a broader term which also encompasses DNA fragments originating from normal non-cancerous cells. The utilisation of ctDNA as tumour biomarker involves identification and quantification of tumour-specific genetic or epigenetic aberrations through DNA sequencing along with the evolving field of cfDNA ‘fragmentomics’ which focuses on the fragmentation pattern (60). The exact modality of ctDNA analysis – for example mutation or methylation detection – and the role of ctDNA in NEN management are yet to be fully established.

The diagnostic utility of ctDNA has been explored in work by Boons *et al.* (26) who used shallow whole-genome sequencing of ctDNA to demonstrate that copy number profile can be used to distinguish between pancreatic NET and adenocarcinoma. Furthermore, Zakka and colleagues applied the Guardant 360® ctDNA panel to 320 patients with NEN and found genomic alterations in 87.5% of samples (24). Differential variant patterns were noted between NEC and NET ctDNA, in keeping with tumour genotyping studies which indicate a higher frequency of mitogen-activated protein kinase (MAPK)-related mutations in NEC versus NET (25). Detection of these variants has potential clinical utility to resolve the diagnostic issue of distinguishing grade 3 gastroenteropancreatic NET from NEC.

An important application of ctDNA analysis in NENs is for detection of therapeutically actionable driver mutations and selection of precision treatments or clinical trial eligibility. A study genotyping cfDNA and matched tissue from patients with metastatic pancreatic NET revealed genomic alterations in 68% of tumour and 66% of cfDNA samples. The most frequently altered genes were consistent with pancreatic NET, including *DAXX* (28%), *TSC2* (24%), *MEN1* (24%), *ARID1B* (20%), *ARID1A* (12%), and *ATRX* (12%), all of which demonstrated promising tissue-plasma concordance (18). Although many of these gene mutations are not currently of therapeutic significance, the study still reported clinically actionable variants in 32% of participants including a case with an *ETV6:NTRK* fusion. The patient was initially treated with larotrectinib, while at progression, additional cfDNA analysis identified an *NTRK3* G623R alteration, an acquired mechanism of resistance, and the patient was subsequently treated with a second-generation Trk inhibitor that showed clinical benefit. However, such examples of molecularly targeted treatments selected based on tumour profiling in NEN patients are generally uncommon.

Aside from somatic mutation detection, DNA methylation profiling of cfDNA has also been explored as a diagnostic method for NET patients. Herrgott *et al.* (61) employed Illumina EPIC array methylation analysis to evaluate differentially methylated regions (DMRs) in the cfDNA of patients with pituitary NET compared with other pituitary lesions (including craniopharyngiomas, pituicytomas, and pituitary cysts) and other central nervous system diseases (including lower-grade gliomas and skull-base meningiomas). These DMRs were used to train a random forest classifier to distinguish between pituitary NET and non-pituitary NET conditions with up to 100% accuracy. Meanwhile, cfDNA hypomethylation and DNA integrity have been explored as markers of NET disease burden and prognosis (62). Such findings require validation; however, they are proof-of-principle for the potential diagnostic utility of cfDNA methylation analysis.

Limitations of ctDNA and cfDNA analysis in the routine care of NEN patients may include a generally low level of ctDNA detected among patients with well-differentiated NETs and low mutation load when compared with other cancer types, increasing the risk of false negatives particularly in low-grade, low-volume tumours. In one study of patients with pancreatic NET, tumour-specific variants could only be detected among those with metastatic disease and not those with localised disease (26), which perhaps limits application for early disease detection. In another study, molecular response in ctDNA following chemotherapy was associated with longer progression-free survival among patients with NECs of gastroenteropancreatic or of unknown primary (63), but ctDNA did not appear to harbour any prognostic utility in another study of patients with low-grade NET (27). One potential benefit of ctDNA for functional phaeochromocytoma or paraganglioma is non-invasive sampling of a patient’s tumour DNA to avoid the risk of hypertensive crisis from a tissue biopsy, which may be of particular use for the surveillance for DNA features associated with acquired drug resistance (64).

### Circulating tumour cells (CTCs) and extracellular vesicles (EVs)

Tumours release cells into circulation as part of the metastatic process, culminating in distant seeding of tumour cells into non-malignant tissues. Therefore, the presence of CTCs is typically a marker of more aggressive metastatic disease and is associated with a poorer prognosis. Several technologies have been developed to isolate and enumerate CTCs, the most established of which is the CellSearch platform which uses an antibody-based approach to select epithelial cell adhesion molecule (EpCAM)-positive CTCs. CellSearch is FDA-approved and validated as a prognostic test in breast, colorectal, and prostate cancers, although it is not widely used in the real-world clinical setting (65).

In a prospective study of 176 patients with metastatic NET, 49% of patients had detectable CTCs via CellSearch. The presence (i.e. at least one CTC) versus absence of CTCs was determined to be the optimal prognostic threshold with respect to progression-free survival (hazard ratio (HR) 6.6, *P* < 0.001) over a median follow-up period of 12.6 months and was also associated with reduced overall survival, high tumour grade, and higher tumour volume (28). More recently, 199 patients with metastatic pancreatic or midgut NET were followed for a minimum of 3 years or until death. The optimal CTC threshold for predicting progression or death was ≥1 for pancreatic NET and ≥2 for midgut NET. Using these thresholds, the odds ratio (OR) for progression at 12 months and death at 36 months, respectively, was 6.69 (*P* < 0.01) and 2.87 (*P* < 0.03) for pancreatic NETs, and 5.88 (*P* < 0.003) and 5.09 (*P* < 0.005) for midgut NETs (29). Other studies have also suggested a prognostic correlation for CTCs in NET; however, scepticism has been raised regarding its clinical utility due to its lower reliability, sensitivity, and specificity compared with other biomarker types (38, 66).

Beyond quantification, isolation and analysis of CTCs can enable genomic or metabolomic analysis, providing an alternative when standard tissue biopsy is not achievable. Indeed, Schlafen 11 (*SLFN11*), an emerging marker of sensitivity to DNA-damaging cytotoxic chemotherapies, has been detected in CTCs of patients with small cell lung cancer, with interval changes corresponding to treatment response (67). Moreover, evolving techniques which allow the capture and analysis of CTC clusters may yield greater biological insights than that of single CTCs (68). However, given the indolent nature of most NETs (69) along with the relatively low CTC levels detected thus far even among patients with advanced metastatic disease (28, 70, 71), it is likely that the majority of NETs in real-world clinical settings are low CTC shedding, thereby limiting the overall utility of this approach.

EVs are small lipid bilayer-enclosed particles released from cells that carry a range of biomolecules and play an important role in cell-to-cell communication. There is a growing interest in the diagnostic potential of EVs for cancer patients (72). To date, there have been few studies on EVs relating to NET patients, but one study reported an increased number of small EVs in the blood plasma of pancreatic NET patients, including grade 1 disease compared with healthy controls, highlighting a potential diagnostic application (30). Both CTCs and EVs represent early investigational biomarkers in NENs; however, further exploration and validation are required before clinical use.

### Proteomics and peptidomics

The high complexity and dynamic range of the circulating blood proteome has historically posed major analytical challenges; however, innovations in instruments, multiplex workflows, acquisition techniques, and data processing have resulted in two competing approaches which enable high-throughput proteomics with unprecedented precision and analytical depth (73). Proteomic biomarkers of NEN may be more promising than ctDNA or CTCs in indolent, low-grade disease due to the inherent secretory function of neuroendocrine tissues as well as less potential to be impacted by low tumour shedding (74).

Affinity-based approaches use engineered affinity reagents (e.g. antibodies or aptamers) to capture specific proteins for digital quantification. Current platforms such as the Olink Proximity Extension Assay (PEA) and SomaScan 11 k/Illumina Protein Prep (IPP) have a readout of >5.4 k and >9.5 k unique proteins, respectively (75). Meanwhile, recent advances in mass spectrometry have significantly increased the throughput of this technology enabling blood-plasma proteomics discovery. Furthermore, optimisation of sample pre-processing for mass spectrometry allows either depletion of high‐abundance proteins (e.g. albumin) or functionalised nanoparticles to reduce the huge dynamic range of blood plasma proteins, enabling detection of low-abundance cancer-related proteins (73).

The application of contemporary proteomic techniques in NEN research is limited, although studies to date have yielded promising results. Using Olink PEA, the EXPLAIN trial identified a panel of 92 proteins which differentiated pancreatic NET patients from controls with a sensitivity of 0.71, specificity of 0.97, and AUROC of 0.99 despite only 57% of the NET patients having an elevated CgA (31). Moreover, this panel was able to distinguish patients with pancreatic versus small intestinal NET with a sensitivity of 0.43, specificity of 0.96, and AUROC of 0.96 (31). In addition, the tyrosine‐protein kinase Lyn was identified as important in identifying pancreatic NET and represents a potentially actionable drug target with the existing Lyn-inhibitory tyrosine kinase inhibitors dasatinib, nintedanib, and bosutinib (31). Another study recently documented 187 proteins associated with metastatic duodenopancreatic NET via mass spectrometry-based proteomic profiling among an international cohort of 56 MEN1 patients with and without disease (32).

Studies evaluating the clinical translatability of these technologies remain limited, and it is still unclear which platform is best suited for detection of cancer biomarkers in blood plasma. Comparative data indicate variable correlation of individual proteins between proteomics platforms in part due to differential interactions of method-specific reagents with protein isoforms, epitopes and/or conformation, or interference via other circulating molecules (76, 77, 78), thus potentially limiting the translatability of results across platforms. Moreover, serial assay expansions for targeted platforms can alter performance and necessitate continued comparative and validation studies. It remains to be established whether one platform is preferred in certain clinical or research scenarios. For example, Olink PEA has indicated superior correlation to independent assays, including ELISA (76, 77) and targeted mass spectrometry (78), when compared with SomaScan; however, SomaScan demonstrated a lower coefficient of variance in repeated measures and greater analytical breadth (77).

### Metabolomics

The assessment of circulating catecholamine catechol-O-methyltransferase (COMT) metabolites (in the form of plasma metanephrines measured using LC/MS) forms a central pillar of non-invasive phaeochromocytoma and paraganglioma assessment. Plasma metanephrines are used for screening, diagnosis, prognostication, and post-treatment surveillance, recognising the limited utility in the small minority of non-functioning phaeochromocytomas and paragangliomas (4). Metabolomics can also elucidate the underlying pathogenic variant of phaeochromocytomas and paragangliomas due to their unique genotype-specific pathogenesis and may be of particular clinical utility when genetic testing results are uninformative or yield variants of uncertain significance. For example, relative concentrations of succinate, fumarate, malate, and other Krebs cycle metabolites can be used to determine pseudohypoxic cluster 1A mutations (79); however, this remains to be validated in blood. Meanwhile, other studies have performed plasma metabolomic profiling in patients with lung and gastroenteropancreatic NETs indicating differences between NET patients and non-cancer controls (33, 34), although the specificity of these findings to NET in comparison with other proliferative or inflammatory states is unclear.

## Conclusion

There remains an outstanding clinical need for accurate non-invasive biomarkers for NEN diagnosis, prognostication, and treatment decision-making to inform effective personalised patient care in a rare and oftentimes unpredictable disease. Efforts to identify novel NEN circulating biomarkers are challenging due to the rarity and heterogeneity of disease, a relatively limited understanding of NEN pathogenic mechanisms and predictors of therapeutic response versus resistance when compared with other malignancies, lack of standardised methodology, and formative expertise in translating next-generation omics technologies into accessible and affordable clinical tools. However, with ongoing technological advancement, there is immense potential to develop precise blood-based diagnostics targeting a range of analytes that can revolutionise care for NEN patients through improved clinical outcomes and quality of life, and reduced treatment burden. Future efforts should ensure a coordinated international approach with routine research biobanking embedded within clinical trials to facilitate biomarker discovery and validation. Further investigation of multi-omics approaches (i.e. integrating multiple omics technologies concurrently) and use of machine learning can accelerate understanding of NEN biology and reveal novel biomarkers which improve diagnosis and personalised care. Technologies for point-of-care testing (80) should also be explored to increase accessibility of blood tests in healthcare settings beyond specialised academic centres.

## Declaration of interest

The authors declare that there is no conflict of interest that could be perceived as prejudicing the impartiality of this work.

## Funding

This work did not receive any specific grant from any funding agency in the public, commercial, or not-for-profit sector. ASX is supported by The National Health and Medical Research Council (ROR: https://ror.org/011kf5r70) Postgraduate Scholarship (GO8345), The University of Melbourne Beaney Scholarship in Pathology, and the Peter Mac NET Centre of Excellence PhD Project Grant. 
